# Shear Strengthening of High Strength Concrete Beams That Contain Hooked-End Steel Fiber

**DOI:** 10.3390/ma15010017

**Published:** 2021-12-21

**Authors:** Hyun-Do Yun, Gwon-Young Jeong, Won-Chang Choi

**Affiliations:** 1 Department of Architectural Engineering, Chungnam National University, Daejeon 305-764, Korea; wiseroad@cnu.ac.kr; 2 Korea Land and Housing Corporation, Jinju-si 52852, Korea; ggwon0515@lh.or.kr; 3 Department of Architectural Engineering, Gachon University, Seongnam-si 13120, Korea

**Keywords:** shear, hooked-end steel fiber, span to depth ratio, ductility

## Abstract

Steel fiber has been used successfully in concrete mixtures to control volumetric changes, including shrinkage. However, the feasibility of the use of steel fiber has been restricted to nonstructural construction, such as ‘slab on ground’. Recently, researchers have attempted to expand the applications of steel fiber to replace structural reinforcement (rebar) and have shown promising results in its substitution for shear reinforcement. Few studies have been conducted to ensure the feasibility of using steel fiber in structural components, however. This experimental study was designed to investigate the shear performance of steel fiber-reinforced concrete beams using the tensile strength of steel fiber and the shear span-to-depth ratio as variables. The experimental results indicate that the tensile strength of steel fiber significantly affects the shear strength of steel fiber-reinforced concrete beams, regardless of the shear span-to-depth ratio, and that steel fiber can play a role in shear reinforcement of concrete beams.

## 1. Introduction

Steel fiber-reinforced concrete (SFRC) has excellent mechanical characteristics in terms of shear and flexural strength, impact resistance, and fatigue resistance. Further, local cracks in SFRC can be controlled by redistributing the cracks throughout the concrete matrix. Recently, SFRC has been implemented for various construction uses, including slabs on ground and structural members.

Since the late 1990s, the feasibility of replacing the shear reinforcement in SFRC has been investigated, as have several parameters associated with shear strength of the SFRC beam, such as fiber type, compressive strength of concrete, and steel fiber contents [1,2,3,4,5,6,7,8].

Casanova and Rossi proposed the specific values of the fiber volume fraction of 0.75% for the minimum shear reinforcement replacement [1]. The shear strength of SFRC is greater than 0.3√(f_ck) when the fiber volume fraction is 0.75% or greater and the shear force of the minimum shear reinforcement is more than 1.5 Vc (0.25√(f_ck)). The shear resistance of steel fiber is equivalent when the minimum shear reinforcement is used.

Recent studies confirmed that these limitations are important in determining the performance of SFRC [9]. For example, SFRC can replace the minimum reinforcement in normal strength concrete, as well in high strength concrete [10,11,12,13,14,15,16]. Furthermore, [17] the investigated and evaluated SFRC beams were reinforced with diagonal reinforcement (rebar) at two compressive strength levels (60 MPa and 100 MPa) to apply the structural restraint effect of reinforced concrete beams [17]. The use of SFRC can reduce the restrained reinforcement in coupling beams by 50 percent. Further, the binding force of SFRC is closely related to flexural toughness. However, the toughness of SFRC shows a considerable decrease with an increase in compressive strength, and the fiber volume fraction of the steel fiber must be increased to achieve the same level of flexural toughness. This problem is closely related to fracture energy, which is generated at the initial crack. Importantly, high levels of fracture energy can cause bond failure. 

More studies are needed to obtain improved flexural toughness in high strength concrete. A recent study [18] evaluated the possibility of replacing the minimum shear reinforcement with steel fiber in high strength concrete. The concrete’s flexural strength was increased by the inclusion of the steel fiber, but its deflection and ductility were decreased when fibers were added to the mixture. The failure mode of SFRC beams changed from compressive failure to tensile failure. Nonetheless, the use of steel fiber to replace the minimum shear reinforcement in high strength concrete is feasible.

The fib Model Code 2010 (MC 2010) [19], which is the European concrete structure standard, recommends the structural use of steel fiber based on the results of flexural tests performed according to EN-14651 [20]. Steel fiber can be substituted for some or all tensile rebar when the results of tests performed in accordance with EN-14651 meet two conditions. The American Concrete Institute (ACI) (ACI 318-14) defined the minimum volume fraction of steel fiber that is needed to replace minimum shear reinforcement and specified that the performance of SFRC is based on the flexural toughness test in accordance with ASTM C1609 [21]. However, the current ACI-318 standard does not take into consideration characteristics such as aspect ratio and the tensile strength of the steel fiber, and the compressive strength of concrete is limited up to 40 MPa. 

In short, this study aims to extend the implementation of the use of steel fiber as a substitute for shear reinforcement in concrete beam associated with the shear span-to-depth ratio of SFRC beams and tensile strength of steel fiber.

## 2. Experimental Program

### 2.1. Manufacturing of SFRC Beams

**Figure 1:**
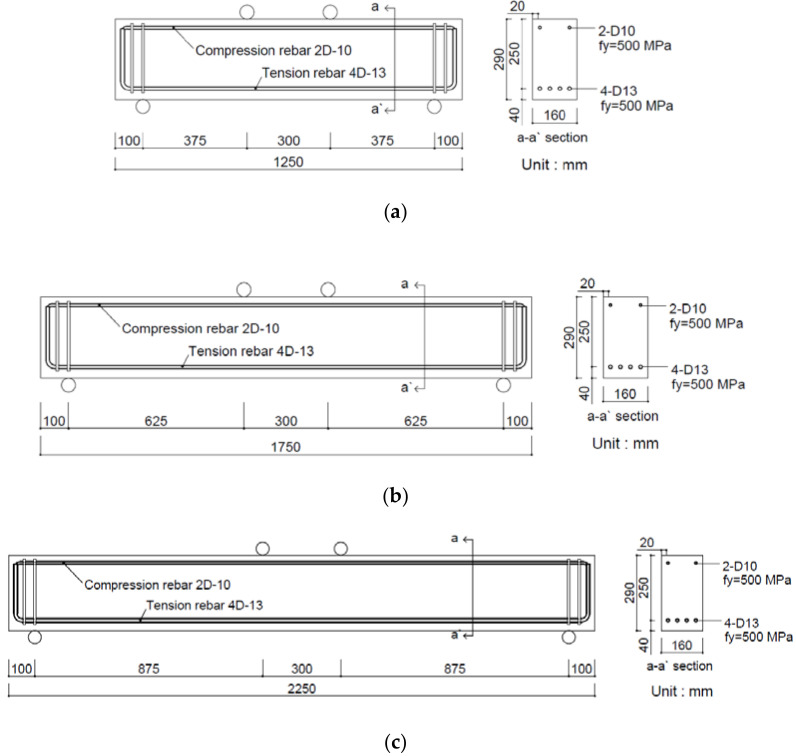
Details of steel fiber-reinforced concrete test beams according to shear span-to-depth ratio (*a/d*). (**a**) *a/d* = 1.5. (**b**) *a/d* = 2.5. (**c**) *a/d* = 3.5.

For this study, six SFRC beams composed of high strength concrete were manufactured according to the tensile strength of the steel fiber and the shear span-to-depth ratio (a/d) of the SFRC test beams. Table 1 presents the mixture designations (IDs) of the specimens and the test variables. The six beam specimens were tested at three shear span ratios: 1.5, 2.5, and 3.5. Tensile rebar with SD500 grade D13 (Seoul, Korea) was used as the reinforcement. Figure 1 presents details of the beam specimens at the three shear span ratios. Shear reinforcement was placed at both ends of the beams to prevent premature failure at the supports. The shear and flexural failures were compared with that of the control member with the shear span ratio of 2.5. The section was designed to observe shear failure, flexural shear failure, and flexural failure according to the shear span ratio.

In order to evaluate the mechanical properties of the SFRC, a cylindrical specimen of 100 mm × 200 mm and a prismatic specimen of 100 mm × 100 mm × 400 mm also were manufactured and cured in accordance with ASTM standards.

### 2.2. Manufacturing of SFRC Beams

The design compressive strength of concrete is 70 MPa. The water/binder (W/B) ratio used in the study is 0.33 and the steel fiber volume fraction is 0.75%, as recommended by ACI standards. Ordinary Portland Type I/II cement (Korea) is used and the maximum aggregate size for coarse aggregate is less than 13 mm. Table 2 presents the mix proportions of the SFRC used in this study.

**Figure 2:**
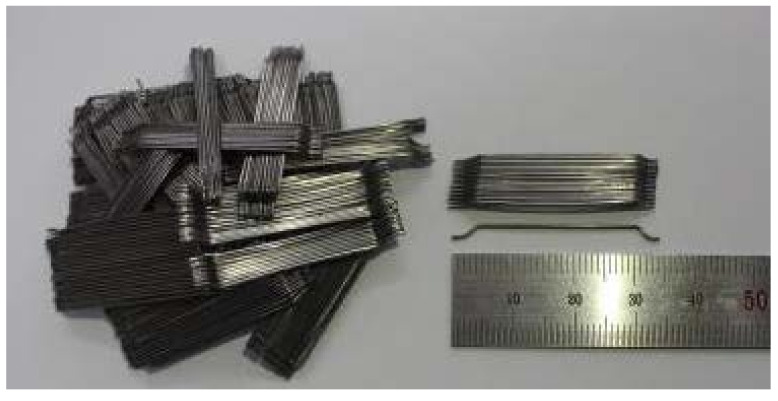
Shape and dimension of steel fiber.

The structural behavior of the SFRC beam specimens was evaluated using both normal and high strength steel fiber. The steel fibers used in this study were the hooked-end type, which Soroushian and Bayasi reported to provide the best performance [9]. Table 3 presents the characteristics of these two types of steel fiber. As shown, the aspect ratio for both types is 64 and the tensile strength levels for the normal strength and high strength steel fiber are 1200 MPa and 1600 MPa, respectively. Figure 2 shows the shape and dimension of steel fiber used in the study.

Two sizes of rebar were used for the test specimens. D10 (SD500) rebar was placed in the upper portion of the specimens for compression reinforcement and D13 (SD500) rebar was placed in the tension side. Table 4 presents the mechanical properties of the two types of rebar used in this study.

### 2.3. Test Set-Up and Instrumentation

**Figure 3:**
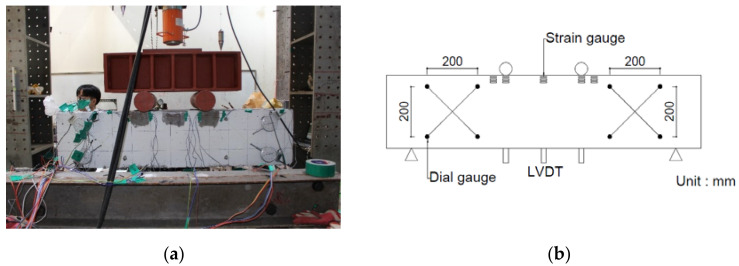
Steel fiber-reinforced concrete beam testing: (**a**) test set-up; and (**b**) gauge installation.

Figure 3a shows the test set-up for the four-point loading that was applied to the SFRC beams to investigate their shear behavior. The shear span lengths of the beam specimens are 375 mm, 625 mm, and 875 mm, corresponding to the shear span ratios of 1.5, 2.5, and 3.5, respectively. A hydraulic jack with 2000 kN capacity was used with load control for the tests. Experimental data were collected using thermal desorption spectrometry, and a linear variable differential transformer (LVDT) was used to measure the deflection of the SFRC beam at the center and at the load points. A strain gauge was attached to the center of the tensile and compression steel rebar to measure the strain of the rebar. Crack size was measured using a microscope at each specified load stage. Figure 3b presents details of the gauge installation lay-out.

## 3. Results and Discussion

### 3.1. Crack Development and Failure Mode of SFRC Beam Specimens

**Figure 4:**
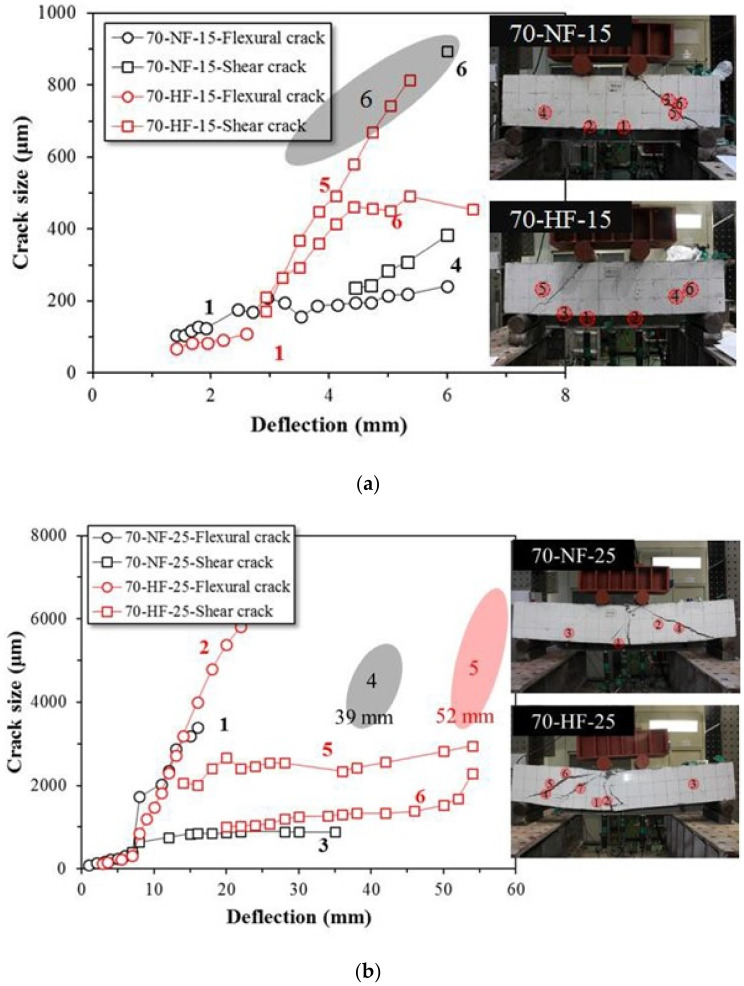

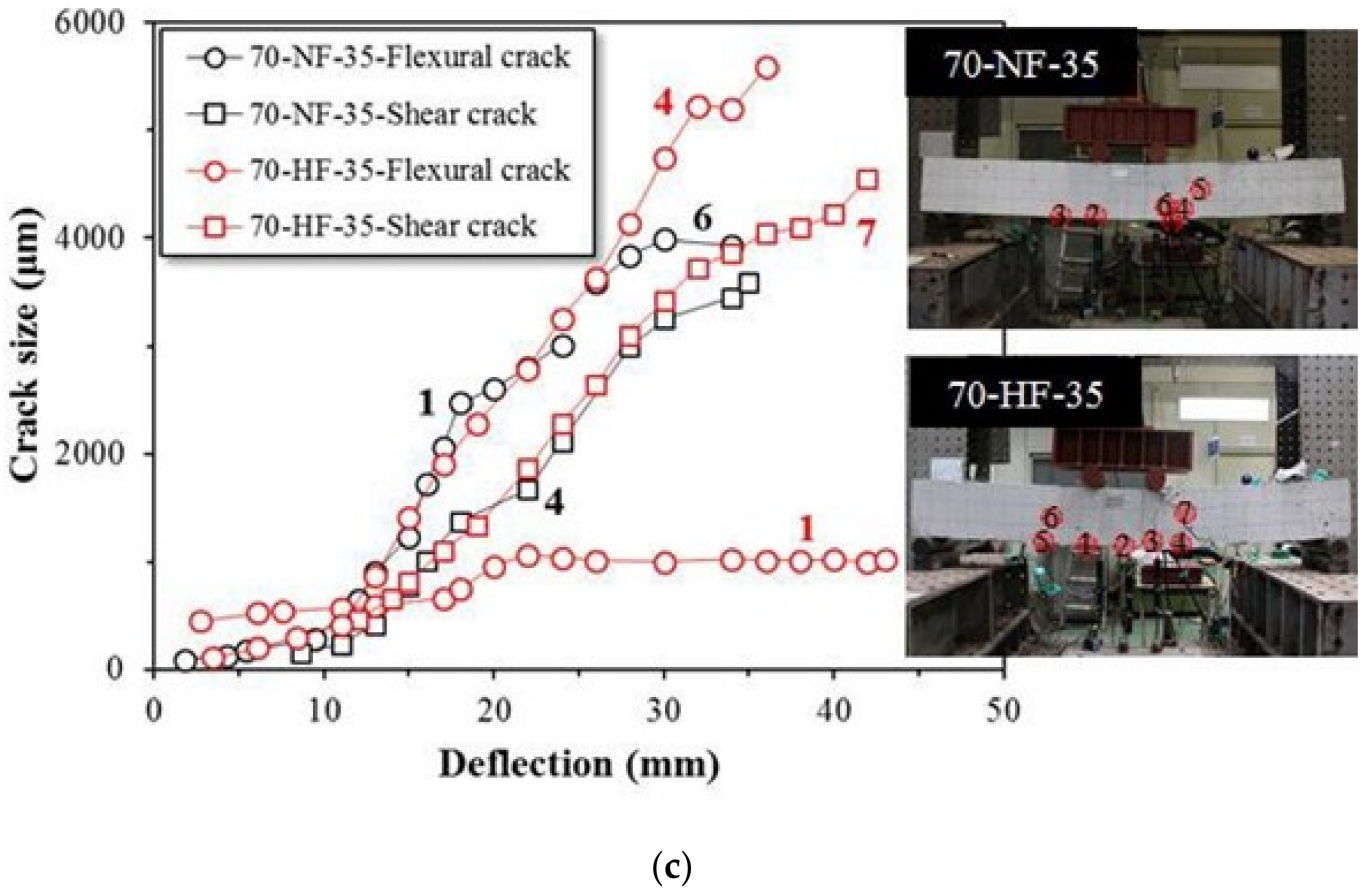
Effects of fiber tensile strength on crack width of steel fiber-reinforced concrete beam specimens at shear span ratios of (**a**) Shear span ratio of 1.5, (**b**) Shear span ratio of 2.5, and (**c**) Shear span ratio of 3.5.

Figure 4a–c shows the effects of fiber tensile strength on the propagation of cracks in the beam specimens at the shear span ratios of 1.5, 2.5, and 3.5, respectively. These figures show the crack size development that was measured by microscope during the tests. For the SFRC beam with normal strength fiber (*a*/*d* = 1.5), the initial flexural crack size is 219 μm, and over 1000 μm of the ultimate crack at failure was measured. For the SFRC beam with high strength fiber, the initial flexural crack size is only 109 μm and the maximum crack is 814 μm at final fracture. For the SFRC specimen with normal strength fiber and the shear span ratio of 2.5, the initial flexural cracks developed up to 3393 μm and shear cracks initiated at the deflection of 39 mm. For the SFRC specimen with high strength fiber, initial flexural cracks developed up to 6528 μm. The test was terminated at the deflection of 52 mm due to the excessive deflection at the midspan of the beam. The maximum crack in the specimen with high strength fiber was larger than the maximum crack in the specimen with normal strength fiber. The crack widened with an increase in the deflection until final fracture even though the load was not decreased by the bridge action of the high strength fiber. For the SFRC beam with normal strength fiber at the shear span ratio of 3.5, flexural and shear cracks developed simultaneously at around 180 μm. However, after the deflection of 15 mm, the flexural crack began to grow rapidly. At the fracture stage, the flexural crack was 3931 μm and the shear crack was 3588 μm. The SFRC specimen with high strength fiber showed a similar tendency whereby the initial flexural crack and shear crack developed at about 60 μm, but the flexural crack grew rapidly after the deflection of 11 mm. At fracture, the flexural crack was 5582 μm and the shear crack was 4555 μm. As expected, shear failure, flexural shear failure, and flexural failure were achieved at the shear span ratios of 1.5, 2.5, and 3.5, respectively.

### 3.2. Load–Displacement Relationship

**Figure 5:**
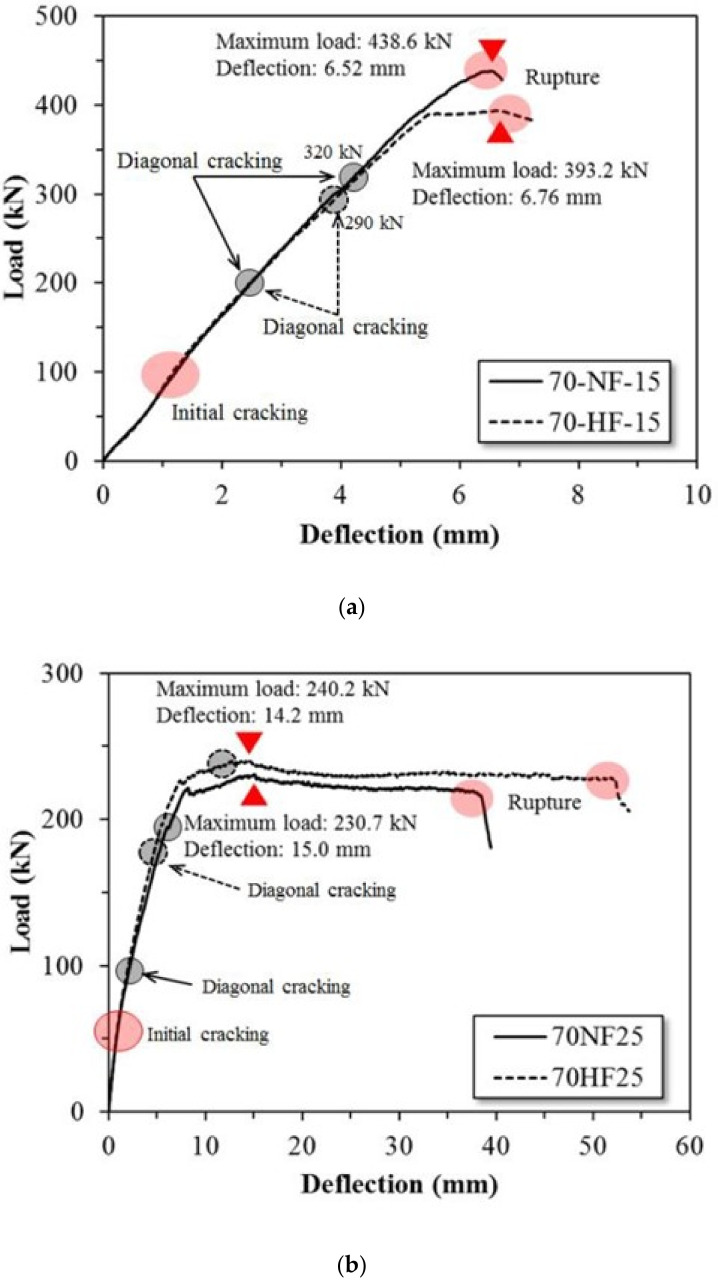

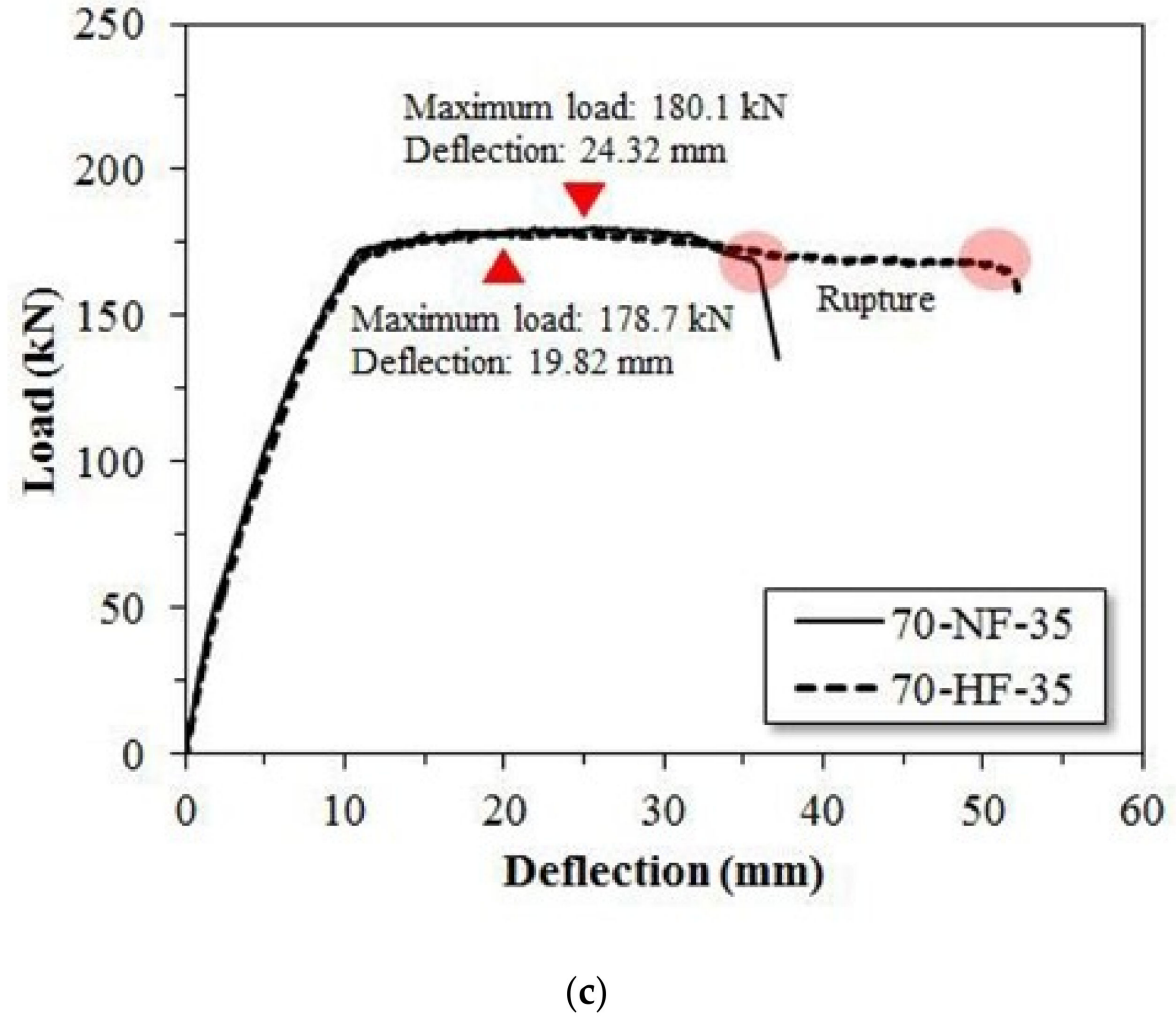
Effects of tensile strength of steel fiber on the load–displacement relationship of SFRC beams. (**a**) *a/d* = 1.5. (**b**) *a/d* = 2.5. (**c**) *a/d* = 3.5.

Figure 5a–c shows the effects of the tensile strength of the steel fiber on the load–displacement relationship of the SFRC beam specimens at shear span ratios of 1.5, 2.5, and 3.5, respectively. For specimen 70-NF-15, initial shear cracks occurred at 110 kN and shear cracks occurred at 200 kN. At 320 kN, diagonal cracks also developed, and shear fracture was induced by the growth of the diagonal cracks. Specimen 70-HF-15 shows a similar tendency toward shear failure mode. Figure 5a shows that the specimens failed in brittle mode at the maximum applied load without any descending portion, regardless of the strength of the fiber. The displacement ductility value (Δu/Δy) is relatively lower than the others, as indicated in Table 5. 

For 70-NF-25, initial shear cracks occurred at 60 kN and shear cracks occurred at 100 kN on the right side of the beam. Diagonal cracks developed on both sides at the deflection of 7 mm, and central flexural cracks developed significantly at the deflection of 31 mm. After the deflection of 38 mm, rapid shear failure was observed due to the development of diagonal cracks. 

For specimens with a shear span ratio of 3.5, initial flexural cracks occurred at the center of the beam at 50 kN and diagonal cracks occurred at 140 kN. From the deflection of 17 mm, central flexural cracks developed continuously, and the beam finally fractured with two flexural cracks. 

For the SFRC beam with high strength steel fiber and the shear span ratio of 1.5, initial flexural cracks occurred at 110 kN. A diagonal crack occurred at 200 kN and an additional diagonal crack occurred at 290 kN. Flexural cracks developed at the deflection of 6 mm and, at the deflection of 8 mm, the diagonal crack widened significantly and shear fracture occurred. 

For the SFRC beams with the shear span ratio of 2.5, initial flexural cracks occurred at 60 kN and a shear crack occurred at 180 kN. At the deflection of 12 mm, flexural cracks and cracks developed on both sides of the beam. Central flexural cracks continued to develop up to the deflection of 48 mm, and two diagonal cracks on the left side of the SFRC beam developed and caused fracture. 

For the SFRC beam with the shear span ratio of 3.5, the initial crack occurred at 50 kN and a diagonal crack occurred at 110 kN. Central flexural cracks and diagonal cracks began to develop at the deflection of 26 mm. At the deflection of 48 mm, the two flexural cracks and diagonal cracks met and finally caused fracture.

Table 5 summarizes the effects of the shear span ratio and fiber tensile strength on the shear and flexural behavior of the SFRC test beams. For the SFRC beam with normal strength steel fiber and the shear span ratio of 1.5, the maximum strength was found to be 438.6 kN at the deflection of 6.52 mm. After the maximum strength was reached, rapid shear failure was observed due to the development of diagonal cracks. When high strength fiber was used, the maximum strength was 393.2 kN at 6.76 mm of deflection. The beam with high strength fiber showed a slight reduction in shear strength compared to the beam with normal strength fiber. However, strength did not decrease after yielding and the beam exhibited ductile behavior. Initial cracking occurred at 100 kN in both SFRC beam specimens.

Initial cracks occurred at 65 kN when the shear span ratio was 2.5. The maximum strength levels were 240.2 kN and 230.7 kN, respectively, according to the tensile strength of the steel fiber. The deflections at maximum strength also are similar. However, in the case of the SFRC beam with normal strength steel fiber, shear fracture by diagonal cracking was observed at 38.55 mm, and the strength decreased rapidly. The maximum deflection was 52.35 mm when the high strength steel fiber was used and increased by 14 mm compared to the SFRC beam with normal strength steel fiber. This outcome is due to the strength of the fiber being greater than the bond strength of the composite and, therefore, is the result of continuous fiber bridge action. 

For the beams with the shear span ratio of 3.5, the experimental results are similar to those for the beams with the shear span ratio of 2.5. In the case of beams with normal strength steel fiber, the strength was found to be 180.1 kN from the deflection of 24.32 mm. The SFRC beam with high strength steel fiber shows 178.7 kN at the deflection of 19.82 mm. The difference in strength according to fiber tensile strength is not significant. However, when high strength fiber was added to the mix, the deflection at final failure was 52.21 mm and the deflection increased by 16.26 mm compared to the case of normal strength steel fiber. This outcome is considered to be the result of continuous fiber bridge action as confirmed by the test results for the shear span ratio of 2.5. In the case of initial crack strength, flexural cracks occurred at the center of both SFRC beams at 50 kN.

### 3.3. Shear Deformation

**Figure 6:**
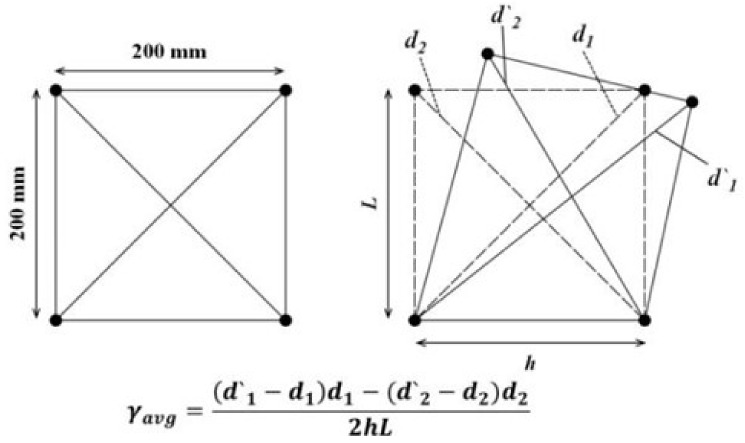
Shear deformation of steel fiber-reinforced concrete beam specimens.

Figure 6 describes the shear deformation of the SFRC beam specimens that was computed using the measured deformation of 200 mm × 200 mm in the X—diagonal direction length at shear span.

**Figure 7:**
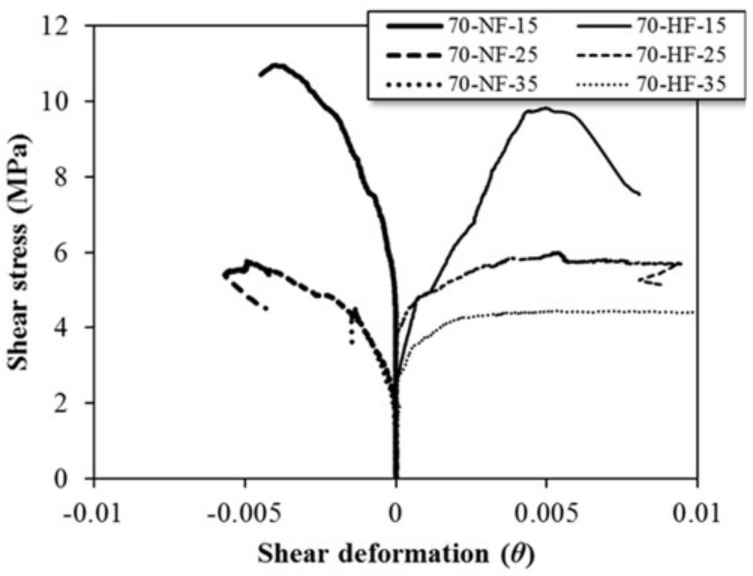
Effect of fiber tensile strength on shear deformation of steel fiber-reinforced concrete beam specimens.

Figure 7 shows the relationship of shear stress versus shear deformation for each SFRC beam specimen. For the specimens with the shear span ratio of 1.5, the shear stress for the specimen with normal strength fiber is less than 0.005 rad, which is greater than the shear stress for the specimen with high strength fiber. A similar trend was observed for all the specimens, regardless of the shear span ratio. These outcomes are the result of the fiber bridge action of steel fiber, which is highly correlated with shear deformation.

### 3.4. Moment–Curvature Relationship

**Figure 8:**
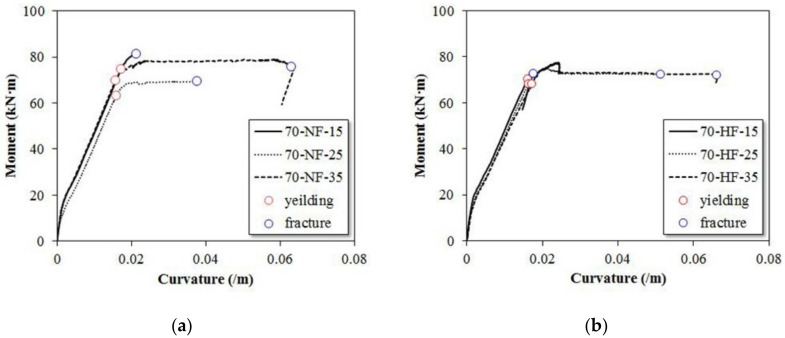
Effects of steel fiber tensile strength on moment–curvature relationship. (**a**) SFRC beam with normal strength steel fiber. (**b**) SFRC beam with high strength steel fiber.

Figure 8 presents the measured strain values for compressive and tension strength and the moment–curvature relationship. The moment–curvature diagram can be divided into three phases. The first phase is linear, which is the phase before the initial crack occurs. After the initial cracking, the curve in the second stage increases nonlinearly with a decreasing slope, which is due to the fact that stiffness does not decrease rapidly after the initial crack initiation but decreases gradually. Specifically, SFRC does not allow the crack width to expand rapidly due to the increase in the load after the initial crack and further microcracks are generated. Therefore, after the initial crack, the crack growth is gradual and, before the maximum moment is reached, a distinct and increasing nonlinear moment period appears. 

The moment curve shows a tendency to decrease gradually due to the steel fiber bridge action after reaching the maximum moment. Figure 8a shows the moment curvature for the SFRC specimens with normal strength fiber. At the shear span ratio of 1.5, the initial stiffness of the two SFRC beams is the same as before the initial cracking, and a slight change in the slope can be observed after the occurrence. The maximum moment and the curvature are smaller/lower than those for beams with normal strength fiber because of the rapid yielding of the high strength steel fiber. At the shear span ratio of 2.5, the maximum moment and curvature are higher when high strength steel fiber was used in the beam. For the shear span ratio of 3.5, similar nonlinear behavior can be observed before and after the initial crack, and the maximum moment and curvature also are similar.

**Figure 9:**
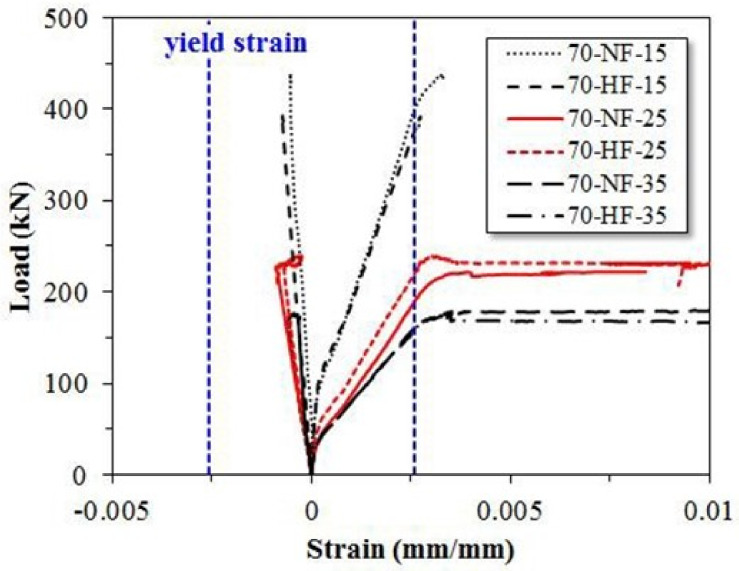
Effects of shear span ratio and steel fiber tensile strength on strain of tension and compression rebar.

Figure 9 shows the measured strain of the central rebar in the SFRC test beams. For the specimens with the shear span ratio of 1.5, the test was terminated before the tension rebar yielded. The compression rebar did not yield either. For the specimens with the shear span ratios of 2.5 and 3.5, the tensile rebar yielded even though the compression rebar did not yield. Due to the tension-controlled behavior, the neutral axis depth is located near the depth of the compression rebar such that the compression rebar hardly deformed.

### 3.5. Shear Strength of SFRC Beam Specimens

**Figure 10:**
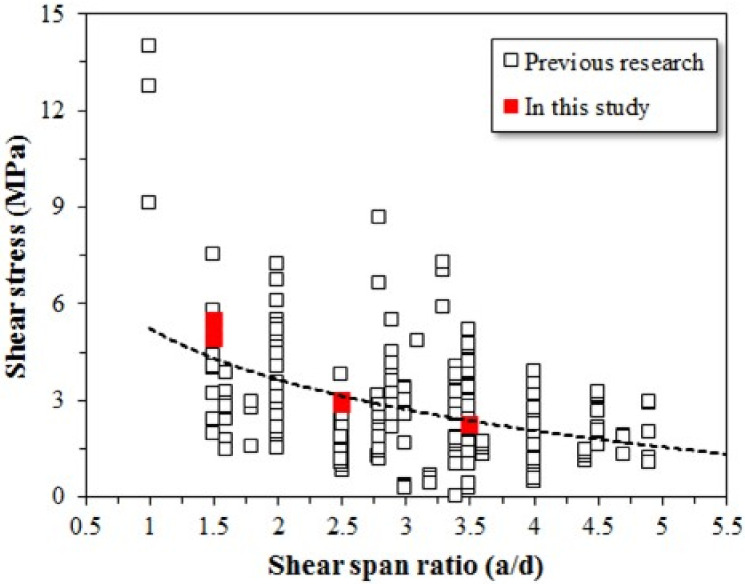
Effects of shear span ratio on shear stress of steel fiber-reinforced concrete beam specimens.

To evaluate the shear strength of the SFRC beam specimens used in this study, 280 experimental datasets were collected from the literature and analyzed. Figure 10 shows the shear stress of the test beams used in this study versus results from other studies for various shear span ratios ranging from 1 to 4.75. The shear strength of the SFRC beams was evaluated according to the tensile strength of the steel fiber; however, no difference in shear strength was evident, regardless of the fiber tensile strength. But, when the fiber tensile strength was increased, ductile fracture behavior was observed after yielding. Ductile behavior was observed at a shear span ratio of 2.5 or higher. Therefore, the effect of high strength fiber on the shear strength of SFRC beams is not significant, but high strength fiber has a significant effect on the ductility of SFRC beams.

## 4. Conclusions

The effect of the inclusion of steel fiber on the shear behavior of high strength SFRC beams was evaluated in this study. Six SFRC beams were experimentally investigated according to their shear span-to-depth ratios. The shear and flexural performance of SFRC beams was evaluated according to the tensile strength of steel fiber and shear span-to-depth ratio with 70 MPa of concrete. When high strength steel fiber was used, the cracks continued to grow even after yielding, showing a ductile behavior. The deflection at final failure increased by 14 mm compared to using normal strength steel fiber. As mentioned in the flexural behavior of SFRC, this is because continuous fiber bridge action becomes possible when the strength of steel fiber is higher than the bonding of the composite. Thus, it was concluded that the deflection capability increased due to the fiber bridge action by high strength steel fiber. The primary conclusion drawn from this study is that the tensile strength of steel fiber does not have a significant effect on the shear strength of SFRC beams, but it does have an effect on ductility. The experimental results confirm the results in the literature. The shear reinforcement can possibly be replaced with high strength steel fiber, and high strength steel fiber shows better performance in high strength concrete than in normal strength concrete.

## Figures and Tables

**Table 1:** Mixture Specimens and Test Variables.

Mixture	Section (mm × mm)	*d* (mm)	Longitudinal Rebar		*_ρ_* (%)	*V_f_* (%)	Fiber Tensile Strength (MPa)	*a/d*
			Bottom	Top				
70-NF-1.5	(*b × h*) 160 × 290	250	4-D13	2-D10	1.27	0.75	1200	1.5
70-NF-2.5								2.5
70-NF-3.5								3.5
70-HF-1.5	160 × 290	250	4-D13	2-D10	1.27	0.75	1600	1.5
70-HF-2.5								2.5
70-HF-3.5								3.5

Note: *d* = depth; *ρ* = reinforcement ratio; *V_f_* = fiber volume; *a/d* = shear span-to-depth ratio.

**Table 2:** Mix Design of Steel Fiber-Reinforced Concrete Mixture.

W/B	Air (%)	Unit Weight (kg/m^3^)					
		W	C	SF	S	G	Steel Fiber
0.33	4	165	475	25	643	813	58.9

Note: W/B = water-to-binder ratio; W = water; C = cement; SF = silica fume; S = sand; G = gravel.

**Table 3:** Characteristics of Steel Fiber Used in Study.

Type	Length (mm)	Diameter (mm)	Aspect Ratio (*l/d*)	Tensile Strength (MPa)
Normal strength steel fiber	35	0.55	64	1200
High strength steel fiber	35	0.55	64	1600

**Table 4:** Mechanical Properties of Rebar Used in Study.

Rebar Size	Yielding Strength (MPa)	Strain at Yielding	Tensile Strength (MPa)	Elastic Modulus (GPa)
D10	473.1	0.00233	637.2	208.7
D13	515.8	0.00257	689.1	200.8

**Table 5:** Effects of Fiber Tensile Strength and Span Ratio on Yield Load and Ultimate Load of Steel Fiber-Reinforced Concrete Beam Specimens.

Mixture	*P_y_* (kN)	Δ*_y_* (mm)	*P_max_* (kN)	Δ*_max_* (mm)	*P_u_* (kN)	Δ*_u_* (mm)	Δ*_u_/*Δ*_y_*
70-NF-15	398.79	5.47	-	-	438.60	6.52	1.19
70-NF-25	221.25	8.27	230.7	15.0	213.76	38.55	4.66
70-NF-35	169.70	9.22	180.1	24.32	166.43	35.95	3.90
70-HF-15	389.65	5.50	-	-	393.20	6.76	1.23
70-HF-25	225.83	7.29	240.2	14.2	225.83	52.32	7.18
70-HF-35	167.41	11.62	178.7	19.82	159.58	48.78	4.20

## Data Availability

The data presented in this study are available on request from the corresponding author.
